# Increase coverage of HIV and AIDS services in Myanmar

**DOI:** 10.1186/1752-1505-2-3

**Published:** 2008-03-14

**Authors:** Brian Williams, Daniel Baker, Markus Bühler, Charles Petrie

**Affiliations:** 1 UNAIDS, 223 Sule Pagoda Road, Yangon, Myanmar; 2 UNFPA, 6 Natmauk Road, Yangon, Myanmar; 3 UNDP, 6 Natmauk Road, Yangon, Myanmar

## Abstract

Myanmar is experiencing an HIV epidemic documented since the late 1980s. The National AIDS Programme national surveillance ante-natal clinics had already estimated in 1993 that 1.4% of pregnant women were HIV positive, and UNAIDS estimates that at end 2005 1.3% (range 0.7–2.0%) of the adult population was living with HIV. While a HIV surveillance system has been in place since 1992, the programmatic response to the epidemic has been slower to emerge although short- and medium-terms plans have been formulated since 1990. These early plans focused on the health sector, omitted key population groups at risk of HIV transmission and have not been adequately funded. The public health system more generally is severely under-funded.

By the beginning of the new decade, a number of organisations had begun working on HIV and AIDS, though not yet in a formally coordinated manner. The Joint Programme on AIDS in Myanmar 2003–2005 was an attempt to deliver HIV services through a planned and agreed strategic framework. Donors established the Fund for HIV/AIDS in Myanmar (FHAM), providing a pooled mechanism for funding and significantly increasing the resources available in Myanmar. By 2006 substantial advances had been made in terms of scope and diversity of service delivery, including outreach to most at risk populations to HIV. More organisations provided more services to an increased number of people. Services ranged from the provision of HIV prevention messages via mass media and through peers from high-risk groups, to the provision of care, treatment and support for people living with HIV. However, the data also show that this scaling up has not been sufficient to reach the vast majority of people in need of HIV and AIDS services.

The operating environment constrains activities, but does not, in general, prohibit them. The slow rate of service expansion can be attributed to the burdens imposed by administrative measures, broader constraints on research, debate and organizing, and insufficient resources. Nevertheless, evidence of recent years illustrates that increased investment leads to more services provided to people in need, helping them to obtain their right to health care. But service expansion, policy improvement and capacity building cannot occur without more resources.

## Background

### The scope of the HIV epidemic

Myanmar is one of South-East Asia's countries hardest hit by the HIV epidemic. At the end of 2005, UNAIDS and WHO estimate that 1.3% (range 0.7–2.0%) of the adult population were infected by HIV [1]. This percentage results in an estimated 360,000 people (range 200,000–570,000) living with HIV. Epidemiological analysis suggests that the HIV epidemic may be levelling off since the early part of the decade [2] (See Table 1).

**Table 1:** HIV Prevalence for selected population groups in Myanmar 2000–2006

	2000	2001	2002	2003	2004	2005	2006
Men with symptoms of sexually transmitted infection	7.1	8	6.5	6	3.2	4.1	4.9
Injecting drug users	62.7	40.9	24.1	37.9	34.4	43.2	42.5
Female sex workers	38.0	33.5	32.3	31.4	27.5	32.0	33.5
Ante-natal care attendees	2.2	2.2	2.1	1.6	1.8	1.3	1.5
Blood donors	1.0	1.1	1.2	1.2	0.8	0.7	0.4
Military recruits	1.4	1.8	2	2.1	1.6	1.3	1.0
Tuberculosis patients						10.3	11.3

(source: Ministry of Health, 2006, unpublished)

An HIV sentinel surveillance system has been in place since 1992. It found that 1.4% of sampled pregnant women attending ante-natal care services were infected with HIV in 1993 [3]. From an initial nine surveillance sites, the system has progressively expanded to 30 sites in 2005 carrying out sentinel surveillance for women receiving ante-natal care and people attending services for sexually transmitted infections. HIV surveillance for specific high-risk groups is also undertaken, including injecting drug users (four sites), tuberculosis patients (nine sites started in 2005) and female sex workers (two sites). The present surveillance systems does not allow for analysis by site as the sample size is too small. Regional differences in the epidemic cannot therefore be further assessed. Protocols are being introduced in 2007 to include men who have sex with men, to add additional sentinel sites for sex workers, to increase sample sizes and to improve sampling methodology [4].

Concerning knowledge, the latest published behavioural surveillance report of the National AIDS Programme [5] contains data for the general population (15–49 years of age) and youth (15–24 years of age) in 2003. Over 90% of the respondents had ever heard about HIV. Knowledge of three effective prevention methods (abstinence, being faithful to one uninfected partner and consistent condom use) ranged from 21% among youth to 42% among the population aged 25–49. The level of knowledge among women of all ages was generally lower than among men. In a 2005 survey on knowledge of reproductive and sexual health the Department of Health Planning surveyed 14,400 households sampled from 86 townships which were part of a UNFPA funded reproductive health programme. It was found that the proportion of the adult respondents (aged 15–49) who could correctly identify at least three ways of preventing HIV transmission was 50.7%. This figure is more than 10% higher than that of a 2002 study by the Department of Health Planning using the same methodology in the same area. [6].

With respect to condom use, in the behavioural survey of the National AIDS Programme, 60% of young men (15–24 years) reported consistent condom use with sex workers [3]. This figure, which some epidemiological models suggest is already high enough to have a significant impact on the spread of the epidemic [7], is largely consistent with data from studies conducted by non-government actors [8]. In an unpublished, national condom market study conducted by Population Services International at the end of 2004, 85.4% of young people (15–24 years) reported condom use the last time they had sex with a sex worker. Another unpublished NGO study in 2004 among youth 15–24 years old and living in Kayin and Mon states found that 82% reported condom use at last sex with a sex worker. Other non-governmental service providers are known to also collect behavioural data for programme monitoring and evaluation purposes but these remain unpublished as official approval for publication has not been sought or granted.

### Support to National AIDS Planning, Coordination and Resource Mobilization

The national response to HIV and AIDS was slow to take off during the 1990s, despite increasing evidence that HIV prevalence was rising. A number of factors constrained the range of services available for HIV activities during the first ten years of the epidemic. Myanmar has an under-funded public health system and limited political support was expressed in support of HIV services. There were few national civil society organisations with HIV programmes, and the formation of civil society in general, outside of those linked to the government, remains problematic. Among the limited number of international non-governmental organizations present in Myanmar, a few started HIV prevention programmes on a limited scale after 1995 and initiated critical advocacy work. UNICEF began supporting services for HIV as early as 1994. As one of the few donors present in Myanmar during that period, UNICEF supported a range of interventions in HIV prevention. The World Health Organisation (WHO) provided training and technical assistance for HIV surveillance, the management of sexually transmitted diseases and the prevention of mother to child transmission of HIV. The United Nations Development Programme (UNDP) provided support to the National AIDS Programme as well as local civil society organisations. Activities supported included condom promotion and supply, provision of test kits to the national blood safety programme as well as the production of information, education and communication materials.

By the turn of the millennium, interest in expanding work in the area of AIDS had grown, but there was no formal mechanism coordinating such efforts. More international NGOs had been able to establish operations in Myanmar and a few parastatal national organizations had begun discussing HIV and AIDS. The National AIDS Programme, though continuing to be based largely around health sector activities, added some non-health sector HIV prevention and awareness-raising work [9], albeit with very limited funding. The Ministry of Health budget for AIDS in 2004, for example, was 78.05 million kyats [10] (this corresponds to $90,000 using the average UN exchange rate for 2004 of 880 Kyats per US dollar) as compared to $1 million in Cambodia, $5.6 million in Viet Nam and $92.8 million in Thailand in 2004 [1].

Early into the new decade, the United Nations agencies present in Myanmar increased their level of investment and began advocating collectively, both within and outside of the country, for increased, concerted action on HIV. A United Nations Joint Action Plan (2001–2002) was developed, and the Joint United Nations Programme on AIDS opened an office. In 2002, a United Nations Expanded Theme Group on AIDS with membership including organisations outside the United Nations system was established and it developed the Joint Programme on AIDS in Myanmar 2003–2005, negotiated with the Government, the National League for Democracy (the leading opposition party) and donors.

The Joint Programme articulated a multi-sectoral framework into which all constituencies (Government departments, United Nations agencies and national and international NGOs) could position themselves and which increased the focus on specific vulnerabilities around the purchase of sex by men and drug use [11]. Technical coordination mechanisms were established. Harmonized indicators were negotiated, providing a basis for collecting annual, comparable data from all partners working on AIDS and assembling a picture of national progress. The United Nations Expanded Theme Group governed the Joint Programme, a body including three representatives from the Ministry of Health, six United Nations agencies, five donors, and three international and three national non-government organisation representatives. While normal practice in many countries, it demonstrated the ability to craft structures in Myanmar, to discuss HIV programme issues and provide a basis for accountable delivery of international assistance.

The Fund for HIV/AIDS in Myanmar (FHAM) was created by three donors – expanded to six by 2006 – to finance the Joint Programme. In the end, the FHAM programmed approximately $26 million over four years starting from 2003, financing the work of 35 implementing partners. UNAIDS Myanmar estimates that the FHAM contributed to roughly 30% of the total funding on AIDS in 2005. The FHAM was itself a product of United Nations collaboration, relying on UNDP to manage the finances and administration of contracts, while the UNAIDS Secretariat mounted a programme support team and chaired a management committee to oversee the use of FHAM funds. The FHAM programme support team monitored all partners' activities on the basis of quarterly progress and financial reports as well as annual reports. During its four years, the Fund undertook a total of 35 field monitoring missions in 62 locations across Myanmar.

### Service delivery expansion: evidence

As a result of the increased investments in AIDS programming, advocacy efforts in favour of a stronger and more coordinated response, and Government steps to improve the enabling environment, prevention and care service provision for HIV grew. By 2005, these investments had started to pay off and significant increases in service provision were reported by implementing partners [8,12,13].

**Figure 1:**
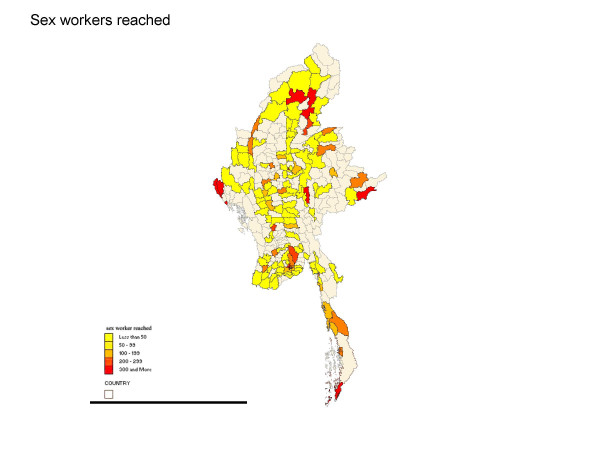
**Number of female sex workers reached by NGO HIV prevention programmes 2005**. Source: National AIDS Programme: **Response to HIV/AIDS in Myanmar: Progress Report 2005**. Yangon 2006.

In 2005, the National AIDS Programme and 15 non-governmental organisations reported reaching a total of 25,500 female sex workers by targeted HIV prevention services. The services were spread over a substantial part of Myanmar with a more concentrated effort in the large urban centres (see Figure 1). Sex work is illegal in Myanmar. The Ministry of Home Affairs issued an unpublished internal directive in 2001 instructing police not to use possession of condoms as evidence of prostitution. More recently, the National Strategic Plan underlines the importance of reaching sex workers in a supportive environment. Unpublished reports of implementing partners highlight the concern of continuing arrests, however.

Drug use is illegal. This poses a number of constraints on programmes addressing the prevention of HIV transmission through contaminated injecting equipment as well as the operation of methadone maintenance programmes. Despite these constraints, current programmes now cover many of the essential elements of a comprehensive harm reduction strategy. The services for injecting drug users had likewise seen a substantial increase. In 2002 only one drop-in centre was in operation; by 2006 a total of 16 drop-in centres, run by NGOs or the United Nations, were operating with high numbers of drug users. In addition to these centre-based services, outreach and peer education teams established in these centres provided prevention and referral services. A total of 11,500 injecting drug users of an estimated total of 60,000 were reported as having received services in 2005 in many of the drug producing areas for Myanmar including Shan and Kachin States, as well as urban centres [8].

Needle exchange and distribution also showed a steep increase in numbers. From 210,000 clean needles distributed in 2003, the reported numbers climbed to 1,162,000 needles distributed in 2005 [8].

Preparation for the roll out of methadone maintenance therapy started in 2004. By the end of 2006 more than 200 people were enrolled in this programme. The methadone programme is implemented in the drug treatment centres of the Ministry of Health. One non-government organisation collaborates with public health services in dispensing methadone. This collaboration between public and non-government sectors is considered crucial to ensure a comprehensive approach in support of patients.

HIV prevention efforts for men who have sex with men are a relatively recent occurrence. Nevertheless, during 2005 at least 22,000 men who self identified as having sex with other men had received tailored health education, mostly through peer education and outreach programmes of non-governmental organizations' [8].

The prevention of mother to child transmission (PMTCT) programme was launched in 2000 by the National AIDS Programme with the assistance of United Nations agencies, and was functioning in 89 out of the 324 townships and 37 state, divisional and other hospitals by the end of 2006. In 2005, a total of 629 mother-baby pairs received Nevirapine (an anti-retroviral drug) prophylaxis through the National AIDS Programme as well as three non-governmental organisations.

The Ministry of Education has introduced life skills training that includes HIV education in the national curriculum for the primary school and in selected secondary schools. The programme has been ongoing since 1998 and the Ministry reports that 46% of the secondary schools are covered by the programme reportedly reaching 900,000 children aged 10 to 16 years in 2005. However, a recent review noted that the quality, coverage and impact of the school-based life skills programme require continued attention [14].

Prevention efforts for specific, targeted groups have been accompanied by advocacy and HIV prevention campaigns for the general population. The mass media have increasingly carried HIV-related message from the government, United Nations agencies and international non-governmental organisations. Population Services International, a non-governmental organisation, reported that 250 HIV-related television spots were shown in 2005. In 2006 this increased to 438. UNAIDS Myanmar tracks HIV media coverage in 10 popular newspapers and journals and found an increasing frequency of HIV and AIDS related reporting since the beginning of 2004 [8].

The availability of condoms either through social marketing or free distribution has increased greatly. With 11.1 million condoms distributed in 1999 compared to 39.9 million by the end of 2005, the figures have risen nearly fourfold over a period of six years [8]. Over half of these condoms were sold at highly subsidized prizes through social marketing, the rest through free distribution. With slightly less that one condom available per capita per year, the figures in Myanmar remain lower than in other South-East Asian countries [8]. These figures do not include commercial sales, roughly estimated as 4.3 million in 2005 by the National AIDS Programme based on informal consultations with partners.

Concerning treatment, care and support, the beginning of anti-retroviral treatment (ART) in Myanmar dates from 2003, when Médecins Sans Frontières Holland first introduced treatment. Since, they have expanded progressively and additional organisations have begun providing treatment, including through the public health sector launched in 2005, resulting in a substantial scale-up (Table 2). Home-based and community-based care has also grown, from 3,800 people living with HIV receiving some sort of support at the end of 2004, growing to 10,900 people at the end of 2005 [8]. A number of self-help groups and networks of people living with HIV have formed over the last years, and there is now representation of people living with HIV in planning events and coordination forums. Further capacity building of localized self-help groups and networks is required, however, to ensure that representatives of people living with HIV have a structure through which they can effectively communicate with their constituents.

**Table 2:** Provision of Anti Retroviral Treatment 2002 – 2006

	**2002**	**2003**	**2004**	**2005**	**2006**
**Number of patients**	17	121	484	2527	5790
**Number of sites**	1	2	10	21	24
Number of organisations*	1	2	4	5	6

Source: National AIDS Programme: *Response to HIV/AIDS in Myanmar: Progress Report 2005*. Yangon; 2006.

* Includes the Ministry of Health's ART programme counted as one organisation.

Access to and uptake of voluntary and confidential counseling and testing remains very low. In order to increase the number of people undertaking HIV testing, provision by an increased number of partners, including NGOs, has been recommended [14]. Recently, two international NGOs have received official permission to launch HIV testing activities.

## Discussion

### Service coverage

The establishment and expansion of AIDS services since 2000 demonstrates that international resources can increase availability of services for populations that would otherwise lack access. In many areas of prevention and care, the number of townships where programmes have been initiated is growing, for example in prevention of mother to child transmission (89 townships in 2006), townships with any kind of sex worker outreach or peer education programme (273 townships in 2005), or townships with HIV programmes for drug users (24 townships) [15]. However, the breadth and depth of service coverage is still alarmingly low when compared to estimated sizes of most at risk populations [16] (see Table 3). Indeed, the number of townships covered does not necessarily translate into significant percentages of people gaining access to services. Less than 20% of injecting drug users are being reached with outreach or tailored health education programmes; in the case of female sex workers this may reach as high as 50% of sex workers, while well under 10% of men having sex with men have access to any service. Only 8% of the estimated number of HIV positive pregnant women is offered services to prevent the transmission of HIV to their babies during birth. Only 10% of people living with HIV estimated as needing anti-retroviral treatment are currently receiving it.

**Table 3:** Coverage of interventions in selected areas of HIV prevention in 2005

	Number reached by services or HIV prevention programs*	Estimated reference population**	**Coverage**
**Female Sex workers**	25,500	40,000	64%
**Injecting drug users**	11,500	60,000	20%
**Men who have sex with men**	22,000	267,000	8%
**HIV positive pregnant women**	629	7,700	8%
**In school youth**	900,000	2,450,000	37%
PLHIV*** receiving ART via public sector + NGOs	5,790	67,000	9%

Sources for reach and estimated populations:

*National AIDS Programme: *Response to HIV/AIDS in Myanmar: Progress Report 2005*. Yangon; 2006.

**Ministry of Health Myanmar: *National Strategic Plan on HIV and AIDS: Operational Plan April 2006-March 2009*. Yangon; 2006.

***PLHIV: People living with HIV.

### Further challenges for program implementation and scaling up

As a result actions by the Ministry of Health and the National AIDS Programme and advocacy by international actors, the environment has allowed actors to expand their work on AIDS. At the same time, the overall operational setting remains unpredictable and constrained, without being broadly prohibitive.

Carrying out health and humanitarian programmes in Myanmar is characterized by a high level of administrative control. Obtaining approvals to establish an organization and a programme – whether national or international – can take a year or more. Memorandums of Understanding with detailed workplans must be negotiated annually down to the township level. Approval by a cabinet-level body is required for every international staff member to be posted in Myanmar. All domestic travel by foreigners requires approval, usually with at least three weeks notice, from both the technical counterpart ministry as well as the Ministry of Defence; foreigners cannot visit projects sites, and not even those under their own direct management, without being accompanied by a government official. Approval for importing commodities is slow to be obtained, and international and national NGOs do not benefit from exemptions provided in other countries for the tax-free importation of vehicles and other project supplies. Much of the procurement funded by international sources has been undertaken by various members of the United Nations system. Difficulties related to coordination of roles and timeliness of procurement have in some instances further delayed programme implementation.

Activities are also constrained by limits of the capacity of the implementers and limits that the national health services can influence other government bodies. The external review of the National AIDS Programme undertaken in April 2006 highlights many of these issues [14]. Capacity for action by non-health ministries, critical for HIV prevention, is also weak. While the Ministry of Health has been successful in mobilizing high level endorsement of its National Strategic Plan, more non-health ministries will have to be mobilized if HIV prevention is to achieve the goal of universal access and be sustainable.

Characteristics of the broader operating environment also hamper, rather than facilitate, HIV prevention and care. Discussion of cultural values and roles, much of which must explore traditional norms about sexual behaviour, often for the first time in the public domain, is essential for sustainable HIV prevention. The meaningful participation of people living with HIV and other civil society actors is essential for such discussions and requires an ability to form self-help groups and formal networks across the country. More research from a variety of viewpoints, including from outside the government, is needed to inform debate which best takes place in an atmosphere of a free exchange of ideas. While such cultural discussion is occurring in the growing (but censored) press, as well as through small informal networks of people living with HIV, its expansion is slow and requires a more conducive environment.

Access to populations in need of services remains difficult and in some cases impossible. Some sensitive border regions, other areas containing large numbers of mobile populations, such as mining camps, and conflict areas are off-limits to international NGOs and United Nations agencies. Some progress has been made, but the HIV epidemic in these areas can only be reliably reversed with full access to all parts of the country.

The operational environment remains difficult to predict. In February 2006, the Ministry of Foreign Affairs, the Ministry of Home Affairs and the Ministry of National Planning and Economic Development, issued new draft guidelines to the international community – United Nations agencies and NGOs alike – for the coordination of organisations undertaking humanitarian work [17]. Partners have raised concerns that a rigid application of these guidelines could compromise their work. The United Nations Resident Coordinator, on behalf of the humanitarian community in Myanmar, sent a letter to the government in March 2006 stating standard humanitarian principles that would be required for successful delivery of assistance to Myanmar.

### Resource constraints

Sufficient and predictable resource flows are critical for planning and service delivery. Government health expenditures in 2005 were reported to be $0.37 per person [18] (using the average UN exchange rate for 2005 of 1,030 Kyats per US dollar against reported 376 Kyats expenditures per person) and the percentage of general government expenditure on health in 2003 was 0.5% of gross domestic product, compared with Thailand 2.0%, Cambodia 2.1% and Vietnam 1.5% [19]. Government investment in health care needs to be dramatically scaled-up if the HIV epidemic is to be rolled back.

**Figure 2:**
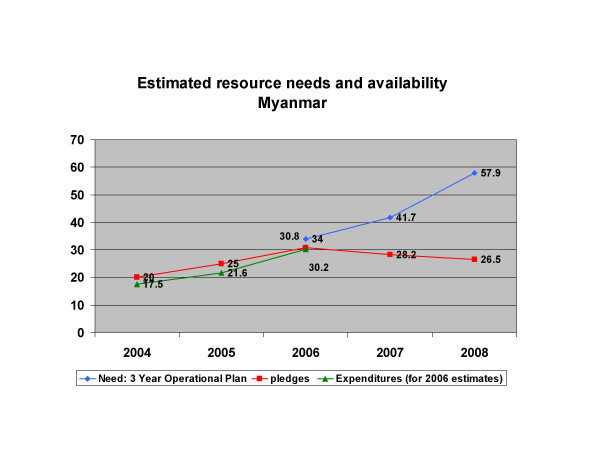
**Trends in resource availability and needs for HIV and AIDS (2004–2008)**. Source: Ministry of Health Myanmar: **National Strategic Plan on HIV and AIDS: Operational Plan April 2006-March 2009**. Yangon 2006. and UNAIDS for 2006 expenditure estimates.

From the international community, Myanmar receives a very low level of financial support considering its development profile. Total official overseas development assistance in the country was estimated as $2.4 per capita in 2004, as compared to $47 for Laos, $35 for Cambodia and $22 for Viet Nam [19]. For HIV alone, in 2005 donor commitments to partners working in Myanmar amounted to approximately $25 million, whereas Cambodia the same year, with a similar epidemic but only a fifth of the population, received approximately $45 million [20]. In 2007, overall resources available for HIV are expected to remain flat (including the anticipated contribution from the three Diseases Fund), handicapping efforts to scale up the response. (see Figure 2).

The highly politicized context of operating in Myanmar requires any potential donor to be very committed to its investment. All grants are closely scrutinized by a variety of political actors both inside and outside the country, who in other circumstances might not pay detailed attention to HIV funding. Donors can expect public commentary on the appropriateness of their investments from the government, the National League for Democracy, Myanmar political groups based outside of Myanmar, and international organizations with a principle focus on political affairs in Myanmar. Large grants can become political issues in and of themselves, testified to by the extensive commentary preceding and following the termination of the Global Fund to Fight AIDS, Tuberculosis and Malaria, Round 3 AIDS grant [21], which occurred in August 2005 for the stated reason that the operating environment did not meet the Fund requirements.

### New Directions in HIV Programming

While partners were slowly expanding services, several motivating – and complicating – factors led to a further evolution of HIV strategic planning and coordination efforts. In line with the "Three Ones" principles being advocated for AIDS programmes worldwide, the government argued for its own leadership role in the national response to AIDS while acknowledging that international standards militated in favour of more participatory practices in strategy design and coordination [22]. An independent mid-term review of the Joint Programme and the FHAM also encouraged the establishment of more complex mechanisms separating out roles of leadership and ownership of national plans, technical support provision by international organizations, and decision-making by investors [23]. Prior to its termination, accommodating the requirements for Global Fund Round 3 also served as motivation for creating participatory coordination structures. The termination in August, 2005, threw planning efforts into turmoil requiring still more adjustment. In early 2006, the government requested an external review of the health sector by a team of international and national experts. The review made a number of recommendations to address the identified short-comings [14].

Reflecting these reviews, events, and evolving views, extensive discussions among all stakeholders led to a new configuration. Continuing the provision of key HIV prevention and care services for the people of Myanmar remained the unifying motivator. The United Nations supported the government in developing a National Strategic Plan 2006–2010 and a targeted, prioritized and budgeted Operational Plan 2006 – 2008 [3,16]. This process involved the government, United Nations agencies as well as international and national NGOs, and was supported by external consultants. Among the advances contained in the new National Strategic Plan include greater coherence among the various actors; a focus on most at risk populations including sex workers and clients, drug users, and men who have sex with men, a participatory coordination structure, more multi-sectoral involvement, an explicit mention of human rights, and a greater emphasis on outcomes (beyond activity outputs) [3]. The Ministry of Health now chairs a Technical and Strategy Group on AIDS which involves representatives from the community of people living with HIV, from other selected ministries, national and international NGOs and United Nations agencies.

Beginning 2006, six donor countries have worked to establish the Three Diseases Fund , responding both to the termination of the Global Fund grants and the imperative to continue the service provision that the FHAM had begun. The Three Diseases Fund's structure more formally divides national strategy making from financial allocation decisions. It provides an incentive for participatory planning and coordination while keeping final decision-making on resource allocation – and the ultimate responsibility for performance – clearly with the donors. It incorporated the United Nations Country Team's statement on principles for the provision of humanitarian assistance into its programme document [24]. It has committed to investing $100 million over five years and will operate through the United Nations Office of Project Services (UNOPS) as its fund manager.

## Conclusion

Since the start of the decade, the provision of HIV prevention and care services has expanded significantly as a direct result of advocacy by internal and external actors concerned about HIV in Myanmar, increased investment of international resources and increased recognition by the Ministry of Health of the issue. Although programme implementation is characterized by high transaction costs and long delays, the environment has not prevented partners from delivering HIV services to people in need but the restrictions have limited geographic coverage and hampered timely implementation. These findings support arguments made as early as 2004 that additional resources can lead to more pragmatic approaches by government [25].

Despite the turbulence created by the Global Fund termination and the generally politicized atmosphere, actors both inside and outside the country have demonstrated that carefully negotiated agreements on HIV and AIDS programming are still possible. The new National Strategic Plan on AIDS 2006 – 2010 currently reflects international best practice in many areas, highlights most at risk populations for HIV, and was developed in a much more participatory manner than any preceding plan. Six donors have crafted an accountable, independent and transparent structure to fund service delivery, using the National Plan as an important reference.

Early indications suggest these new structures offer a way forward in the Myanmar context, yielding benefits for people living with HIV and the population as a whole. Programme output data demonstrates that increased resources and policy engagement can result in increased services for people in need and facilitate the evolution of HIV policies. However, more capacity building of the public health system and NGOs, more operational and behavioural research, more policy discussion, and more partners are all needed to build on this foothold of successful programming. Without more investment, from the Government as well as international sources, the road towards universal access to HIV prevention and care will be much longer than it needs to be.

## List of abbreviations

AIDS Acquired Immunodeficiency Syndrome

ART Antiretroviral Treatment

FHAM Fund for HIV/AIDS in Myanmar

HIV Human Immunodeficiency Virus

NAP National AIDS Programme

NGO Non-governmental Organization

PLHIV People living with HIV

STD Sexually Transmitted Disease

UN United Nations

UNAIDS United Nations Joint Programme on AIDS

UNFPA United Nations Population Fund

UNICEF United Nations Children Fund

UNDP United Nations Population Fund

UNGASS United Nations General Assembly Special Session on HIV

WHO World Health Organisation

## Competing interests

The authors have no competing financial interest. BW, DB, MB and CP are based in Myanmar and work for United Nations agencies. This article was written in a personal capacity and does not necessarily reflect the view of UNAIDS or any other United Nations organisations.

## Authors' contributions

BW and MB led in writing the manuscript. MB also researched background data, prepared tables and charts. DB and CP participated in manuscript preparation.
